# Randomized field trial comparing the efficacy of florfenicol and oxytetracycline in a natural outbreak of calf pneumonia using lung reaeration as a cure criterion

**DOI:** 10.1111/jvim.16348

**Published:** 2022-01-07

**Authors:** Stan Jourquin, Jade Bokma, Lieze De Cremer, Katharina van Leenen, Nick Vereecke, Bart Pardon

**Affiliations:** ^1^ Department of Large Animal Internal Medicine, Faculty of Veterinary Medicine Ghent University Merelbeke Belgium; ^2^ Department of Pathology, Bacteriology and Poultry Diseases, Faculty of Veterinary Medicine Ghent University Merelbeke Belgium; ^3^ Department of Virology, Parasitology and Immunology, Faculty of Veterinary Medicine Ghent University Merelbeke Belgium; ^4^ PathoSense Merelbeke Belgium

**Keywords:** antibiotics, bovine respiratory disease, *Mycoplasma bovis*, precision medicine, rational antimicrobial use, thoracic ultrasound

## Abstract

**Background:**

Respiratory infections are the main indication for antimicrobial use in calves. Optimal treatment duration currently is unknown, but shorter duration would likely decrease selection for antimicrobial resistance.

**Hypothesis/Objectives:**

Determine differences in cure rate and healing time between animals treated with florfenicol and oxytetracycline in a natural outbreak of respiratory disease using reaeration observed on thoracic ultrasound examination as healing criterion.

**Animals:**

Commercial farm housing 130, 3 to 9 month old Belgian blue beef calves.

**Methods:**

Randomized clinical trial during an outbreak of respiratory disease. Metaphylactic treatment was initiated, randomly treating animals with either florfenicol or oxytetracycline. Ultrasonographic follow‐up was done the first day and every other day for a 14‐day period. At the individual animal level, treatment was discontinued when reaeration of the lungs occurred. Differences in cure rate and healing time were determined.

**Results:**

Of the 130 animals studied, 67.7% developed a lung consolidation ≥0.5 cm. The mean ultrasonographic healing time was 2.5 days in the florfenicol group compared to 3.1 days in the oxytetracycline group (*P* = .04). After single treatment, 80.6% and 60.3% had no consolidations in the florfenicol and oxytetracycline groups, respectively (*P* = .01). A *Mycoplasma bovis* strain was genetically and phenotypically determined to be susceptible to both antimicrobials.

**Conclusions and Clinical Importance:**

Ultrasonographic lung reaeration shows potential as a cure criterion to rationalize antimicrobial use for outbreaks of pneumonia. In our study, florfenicol resulted in a faster cure and higher reduction in antimicrobial usage than did oxytetracycline.

**Abbreviations**
ADD: animal daily dosage
BCV: bovine coronavirus
BRD: bovine respiratory disease
BRSV: bovine respiratory syncytial virus
BVD: bovine viral diarrhea virus
DOX: doxycycline
ECOFF: epidemiological cutoff
EMA: European Medicines Agency
ENRO: enrofloxacin
FF: florfenicol
GAM: gamithromycin
GEN: gentamycin
HR: hazard ratio
IBR: infectious bovine rhinotracheitis
MALDI‐TOF MS: matrix‐assisted laser desorption ionization‐time of flight mass spectrometry
MIC: minimum inhibitory concentration
nBAL: nonendoscopic bronchoalveolar lavage
OTC: oxytetracycline
OR: odds ratio
PCR: polymerase chain reaction
PI‐3: bovine parainfluenza‐3 virus
TIA: tiamulin
TIL: tilmicosin
TUS: thoracic ultrasound
TYL: tylosin
qTUS: quick thoracic ultrasound
WHO: World Health Organization

## 1 INTRODUCTION

Respiratory tract infections are a major cause of morbidity, mortality, and antimicrobial use in all cattle industries worldwide, including dairy, beef, and veal farms.^1^, ^2^ Mixed infections are often present, consisting of viruses, *M. bovis* and *Pasteurellaceae* species.^3^ Increasing scientific evidence and consumer concerns regarding antimicrobial resistance selection warrant an urgent reduction in antimicrobial use without hampering animal welfare.^4^, ^5^

Metaphylactic antimicrobial group treatments frequently are used to control bovine respiratory disease (BRD) outbreaks.^6^, ^7^ This approach is increasingly criticized, especially because many outbreaks include viruses and it is unclear to what extent bacterial superinfection occurs.^8^, ^9^, ^10^ A meta‐analysis showed mainly short‐term effects of metaphylaxis and stated that the number needed to treat is too high for this approach to remain acceptable.^8^ However, metaphylaxis likely will remain essential to control some highly contagious bacterial pathogens such as *M. bovis*, which is involved in approximately one third of respiratory disease outbreaks in dairy and beef calves and in almost in every veal calf or feedlot outbreak.^9^, ^11^, ^12^

Thoracic ultrasound (TUS) potentially can reliably identify animals with pneumonia (either clinical or subclinical) and differentiate them from those with upper respiratory tract infections.^13^, ^14^, ^15^ In addition, individualization of treatment length based on TUS may substantially decrease antimicrobial use, even when metaphylaxis is used. In practice, a 5 to 10 day antimicrobial treatment length for pneumonia often is used, but scientific evidence is limited and criteria for success are poorly definded.^16^, ^17^, ^18^ It is unknown whether required treatment duration would differ among antimicrobial classes. Both European Medicines Agency (EMA) and World Health Organization (WHO) recommend more restricted use of macrolides, fluoroquinolones and cephalosporins, whereas florfenicol (FF) and oxytetracycline (OTC) are most frequently mentioned as first choice empirical treatment for BRD in formularies from different European countries.^5^, ^19^, ^20^, ^21^

Historically, criteria for cure have been based on the disappearance of clinical signs linked to respiratory disease.^10^ On TUS, pneumonia is characterized by the presence of ≥1 lung consolidations, and in studies of humans reaeration of a previously consolidated area is considered a sign of healing.^22^, ^23^ It is unknown whether lung reaeration can be used as a criterion to discontinue antimicrobial treatment in cattle.

Therefore, our objective was to assess the possibility of using lung reaeration as an individual animal cure criterion to discontinue antimicrobial treatment. Second, differences in cure rate and required treatment duration between FF and OTC were determined. As a third objective, susceptibility of the involved *M. bovis* strains was determined both by microdilution and genome sequencing.

## 2 MATERIAL AND METHODS

### 2.1 Study design, animals, and housing

The trial protocol was approved by the Ethical committee of the Faculty of Veterinary Medicine and Bioengineering of Ghent University. To compare the difference in cure rate and healing time between animals treated for *M. bovis* pneumonia with FF and OTC, a randomized clinical trial was conducted. The study was carried out in October 2017 on a commercial beef farm housing 1000 Belgian Blue beef cows, located in Eastern Flanders (Belgium). The respiratory disease outbreak involved 163 Belgian blue beef calves, aged 1 to 9 months. Animals were housed in the same stable (shared airspace) in pens of 5 to 6 animals on straw bedding with an approximate surface of 5 m^2^ per animal. Vaccination protocols for bovine respiratory syncytial virus, bovine parainfluenza virus‐3 (PI‐3), bovine viral diarrhea virus (BVD; Rispoval3, Zoëtis), infectious bovine rhinotracheitis virus (IBR; Bovilis IBR Marker Live, MSD), *Histophilus somni* and *Mannheimia haemolytica* (*H. somni* LKT, Hipra) were carried out according to the respective manufacturer recommendations. Animals were fed milk replacer and concentrates for the first 12 weeks, followed by concentrates and hay post‐weaning. The farm was selected based on an occurring outbreak of disease.

### 2.2 Lung ultrasonography, clinical scoring, and follow‐up

Clinical status of the animals was assessed using the following clinical variables: temperature (fever ≥39.3°C), breathing frequency (tachypnea ≥45 breaths/min), head tilt (present or absent), coughing reflex (present or absent), and apathy (normal or lethargic). Calves were considered clinically ill when ≥1 of these variables was abnormal. Clinical status was only determined at the first visit by 2 experienced veterinarians.

Thoracic ultrasound (TUS) was performed at the first visit and subsequently every other day for the 14‐day study period. A portable ultrasound device (Tringa Linear, Esaote, The Netherlands) with a linear probe (5.5‐7.5 MHz) was used to allow evaluation of large parts of the lung between the intercostal spaces. Sufficient contact was achieved using isopropyl alcohol (70%‐90%) as transducing agent. We used the scanning technique and landmarks of the UGhent quick scan (quick thoracic ultrasonography [qTUS]).^24^ To maximize diagnostic accuracy, scanning was not done at the usual qTUS speed of 1 to 2 minutes per animal. Briefly, the scanning technique consisted of 1 single movement on each side of the thorax starting from the tip of the diaphragmatic lobes to the tip of the cranial lung lobes. In larger calves, 2 movements on each side often are needed to visualize the entire lung. After being positioned parallel with the ribs, the probe is moved cranially from intercostal space to intercostal space, without losing contact with the skin. This position maximizes the lung area visualized and minimizes acoustic shadowing of the ribs. The landmark to initiate the scan for the right side is an image containing the lung dorsally and the liver ventrally (8th‐9th intercostal space). On the left side, (8th‐9th intercostal space) it is an image with the lung dorsally, the spleen in the middle and the rumen ventrally. The second landmark is an image taken at the level of the heart (3rd‐5th intercostal space) consisting of the lung dorsally and the heart ventrally in a 50/50 distribution. This landmark is used to reorient the probe. Next, the probe is advanced cranially under the triceps muscle in this position. In this manner, scanning of the cranial lung lobes (mainly the right cranial lung lobe, but visualization of this lobe from the left side is common) can be done in 1 scanning movement with good visualization. The final landmark, the end point of the scanning technique (left and right), consists of the internal thoracic artery and vein and the most cranial part of the cranial lung lobes. During the scan, as soon as any abnormality (comet tail artifact, pleural irregularity, consolidation) is seen, the probe is halted at that position and gently moved cranially and caudally to expand the area of visualization. This approach increases the odds of detecting a consolidation. Consolidation depth was measured in a dorso‐ventral plane using the grid on the screen of the ultrasound device. Detection of consolidated lung tissue (≥0.5 cm depth) was used as a single criterion to confirm pneumonia. After each examination, the farmer and local veterinarian were informed which animals still required treatment.

Based on the combination of TUS and clinical scoring, animals were classified into 4 groups: healthy (no clinical signs, no consolidation on ultrasound), subclinical pneumonia (no clinical signs, but consolidated lung tissue on ultrasound), clinical pneumonia (both clinical signs and consolidated lung tissue), and upper respiratory tract infection (clinical signs, but no lesions on ultrasound).^25^, ^26^ Categorization of animals into 1 of these groups was done at the first visit to visualize clinical status at the farm level.

After the 14‐day trial period, the clinical course of the animals was followed as part of the herd health services provided to the farm, which did not include systematic ultrasonography at the time of the study. The farmer was asked to report signs of decreased growth or carcass weights if divergent from expected results.

### 2.3 Treatment protocol

Metaphylactic treatment was performed on all 163 animals present at the time of the outbreak. Upon request of the local veterinarian and farmer, calves <3 months old (n = 33) all were treated with OTC to avoid diarrhea and gastrointestinal dysbiosis often associated with prolonged FF use.^27^, ^28^ Only calves >3 months of age (n = 130) were included in the trial and randomly allocated, using a list and the RAND function in Microsoft Office Excel (2016), to 1 of 2 treatment groups: Group 1 (n = 62) received FF (Selectan, Hipra) at a dosage of 20 mg/kg whereas group 2 (n = 68) was treated with OTC (Engemycine, MSD) at a dosage of 10 mg/kg. This list of animals and their respective treatment group was given to the farmer, local veterinarian and 1 of the researchers standing outside the pen. Both persons performing the ultrasound examination were not informed to which test group an animal belonged. No externally visible marks were present on the animals to distinguish treatment groups. Once allocated to a treatment group, animals were treated with the same antimicrobial as the previous treatment at the same dose for as long as the lung consolidation was present. Both drugs were administered IM q48h, according to manufacturer recommendations. Body weight was estimated using chest circumference measurements done at first visit and rounded up to the next 10 kg. A consolidation depth ≥0.5 cm was used as a threshold to initiate antibiotic treatment. During the trial period, animals were scanned every other day, only retreating calves with lung consolidations with a depth of ≥0.5 cm. Reaeration was defined as the reappearance of reverberation artifacts (A‐lines) in previously consolidated lung tissue. When complete reaeration of the lung occurred, treatment was discontinued.

### 2.4 Sampling and laboratory diagnosis

Before treatment was initiated, a small‐volume (30 mL saline) nonendoscopic bronchoalveolar lavage (nBAL) was performed in 5 standing, unsedated calves by a veterinarian, using a sterilized catheter as previously described.^29^ Sampled calves were conveniently selected, only assuring they originated from a different pen and showed lung consolidations on TUS. Samples were taken before treatment was initiated. Bacterial cultures for *M. bovis* were done on all 5 samples using a selective indicative agar (Pleuropneumonia‐like organism agar with Tween‐80 for lipase activity), as described elsewhere.^30^, ^31^ Multiplex real time‐PCR (qPCR) for various BRD pathogens was performed on a pooled sample from the 5 nBAL's.^32^ Definitive identification of *M. bovis* was performed using MALDI‐TOF MS (Brüker Daltonik GmbH, Bremen, Germany), as described by previously and qPCR.^32^

Antimicrobial susceptibility of *M. bovis* for both FF and OTC was determined using broth microdilution, determining minimum inhibitory concentration (MIC) using previously described techniques.^33^ Because of the lack of clinical breakpoints for *M. bovis*, MIC values were interpreted by determination of the epidemiological cutoff (ECOFF) using the visual method as described previously.^33^ The ECOFF values for FF and OTC were reported as >16 and >8, respectively.^33^ Strain typing of *M. bovis* and detection of possible genomic antimicrobial resistance was done by long‐read nanopore sequencing using techniques previously described.^34^, ^35^ Both MIC determination and sequencing were performed on an isolate of *M. bovis* from a calf showing both consolidations on TUS and clinical signs of respiratory disease.

### 2.5 Statistical analysis

All data was entered in a spreadsheet (Excel, Microsoft, Inc) and transferred to both SPSS statistics 27 and SAS 9.4 (SAS Institute, Inc, Cary, North Carolina). Graphs were made using GraphPad Prism (version 9.1.1 for Mac, GraphPad Software, San Diego, California, www.graphpad.com). The individual calf was defined as an experimental unit. Primary outcomes of interest were cure rate and healing time. Cure was defined as showing complete reaeration (reverberation artifacts) of a previously consolidated lung. Similarly, cure rate was defined as the proportion of calves that showed lung reaeration of a previously consolidated lung area. Healing time was defined as the number of days between start of the treatment and appearance of complete lung reaeration, as a criterion for lung healing at which antimicrobial treatment was stopped. Healing time represented the minimum required treatment duration to obtain complete lung reaeration.

In this trial, all animals were considered at risk for development of pneumonia. Therefore, for animals that did not show lung consolidations at the first examination (day 1), the healing duration was set at 2 days because the dosing regimen ensured an effective antimicrobial blood concentration for 2 days after metaphylactic treatment was initiated. Because the use of metaphylaxis also relies on the prevention of developing pneumonia, these animals were counted as part of the total number of healthy animals after treatment. All analyses were done for the entire group (animals with and without lung consolidations on the first day of the trial) and separately for the animals with lung consolidations on day 1. Considering a noninferiority limit of 10%, the available sample size allowed for detection of a 10% difference in cure rate between the 2 groups with 80% power and alpha <.05. Using an expected SD in healing time of 4 days allowed for detection of differences in healing time of 2 days between FF and OTC.

To determine the difference in cure rate, multivariable logistic regression was used with cure as binary outcome variable. The overall test for treatment effect was based on the Chi‐squared test (Wald test) and treatments were compared pairwise using Tukey's multiple comparison technique. Each model contained treatment group, time, and interaction of treatment group with time. Pen was forced as a random factor in each model to account for clustering of calves in a pen. In a multivariable analysis, in addition to treatment group sex and clinical signs (temperature, breathing frequency, cough, and apathy) also were evaluated for potential confounding effects on cure.

To bring healing time into account, a Cox proportional hazards model was constructed with cure (0/1) as the outcome variable and treatment group as predictor in a second approach. The time variable was defined as the number of days between initiation of the treatment and discontinuation of treatment when lung reaeration was seen. Right censoring was done at day 14 when the study was ended. The same predictors as mentioned for logistic regression were included.

A linear mixed model was used to determine the difference in required treatment duration until reaeration (i.e., healing time) between FF and OTC. The outcome variable was treatment duration, based on therapeutic blood concentrations (2 days at the long‐acting dose used in the study) and the predictor was treatment group. Again, pen was forced as a random factor into the model. Finally, the number of animal daily dosages (ADD) was calculated for each treatment group by adding the number of days that each animal had received antimicrobials, taking the long‐acting effect (2 days) of FF and OTC into account. One‐ sample t testing was used to compare ultrasound‐guided treatment to a hypothetical 7‐day metaphylaxis. A similar linear mixed model approach as mentioned above was used to determine the difference in ADD between the FF and OTC group. In all models, significance was set at *P* < .05.

## 3 RESULTS

### 3.1 Animals and outbreak characteristics

To avoid age bias, animals <3 months of age were excluded from the study, leaving 130 animals for analysis. At the time of the first visit, 115 animals (88.5%) showed at least 1 abnormal clinical variable, whereas 53.1% (69/130) exhibited ≥2 abnormal variables and were considered clinically ill. Increased temperatures were present in 103 (79.2%) animals, and a positive cough reflex and tachypnea were present in 17.7% (23/130) and 51.5% (67/130), respectively. None of the enrolled calves had a head tilt. At that time, 67.7% (88/130) and 40% (52/130) showed lung consolidations on TUS ≥0.5 and ≥1 cm in depth, respectively. Mean consolidation depth on day 1 was 1 cm (SD, 1; minimum, 0; maximum, 4; median, 0.5). Based on the combination of clinical scoring and qTUS, 16.2% (21/130) were healthy, 38.8% (40/130) had subclinical pneumonia, 36.9% (48/130) had clinical pneumonia and 16.2% (21/130) had upper respiratory tract disease.

### 3.2 Cure rate

**FIGURE 1:**
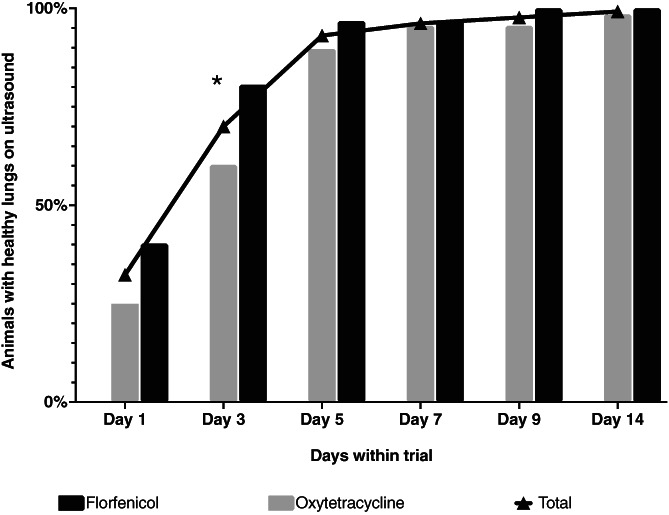
Overview of 130 calves at risk for development of pneumonia during a natural outbreak of respiratory disease. Animals were treated either with florfenicol (n = 62) or oxytetracycline (n = 68), over a 14‐day observation period, using reaeration of consolidated lung tissue on ultrasound as cure criterion. (*) signifies a significant difference in cure rate between both antimicrobials

Cure rates for both treatment groups for the entire study period are shown in Figure 1. Two days after the first injection (day 3), 91 animals showed no consolidation on TUS (70%). When considering all animals at risk, the odds of having no consolidation at day 3 were significantly higher (odds ratio [OR], 2.7; 95% confidence interval [CI], 1.2‐6.1; *P* = .01) in the FF group when compared to the OTC group, showing cure rates of 80.6% and 60.3%, respectively. In the animals with consolidations ≥0.5 cm on day 1 (88/130), cure rate after first injection was 67.6% and 47.1% in the FF and OTC groups, respectively (OR, 2.3; 95% CI, 1.0‐5.7; *P* = .06).

Five days after the first visit, the overall cure rate was 93.1%, with 9 animals (6.9%) having lung consolidations on ultrasound examination. The odds of having lung consolidations ≥0.5 cm were no longer significantly higher in the FF group compared to the OTC group (cure rate of 96.7% and 89.7%, respectively; *P* = .13). In the FF group, 100% cure was reached on day 9, whereas 3 animals in the OTC group still had consolidations indicating active pneumonia. At the end of the trial (day 14), a final overall cure rate of 99.2% was reached, leaving a single chronically ill animal in the OTC group. Pen effect, as well as sex and clinical signs collected on day 1, were not significantly associated with cure rate in the multivariable analysis. The Cox regression analysis showed that the hazard ratio (HR) for cure was 1.3 times higher (95% CI, 0.9‐2.03) in the FF group as compared to the OTC group, but was not significant (*P* = .2). During a period of 3 months after the trial, the farmer did not report any animals showing signs of clinical relapse or growth results under expectations.

### 3.3 Healing time and total number of animal daily dosages

Considering all animals, mean healing time was 2.5 days (SD, 1.3; minimum, 2; maximum, 8; median, 2) and 3.1 days (SD, 2; minimum, 2; maximum, 14; median, 2) in the FF and OTC group, respectively (*P* = .04). One animal did not heal within the observation period and therefore was excluded when calculating healing time. This animal was treated for 1 month, corresponding with an ADD of 30 days. When only considering cases with lung consolidation on day 1 (n = 88), mean healing time was 2.9 days (SD, 1.5; minimum 2; maximum 8; median 2) in the FF group and 3.5 days (SD, 4; minimum, 2; maximum, 14; median, 4) in the OTC group (*P* = .1). When comparing ADD between both treatment groups, a difference of 1 day, with a mean of 2.5 days for FF (SD, 1.3; minimum, 2; maximum, 8; median, 2) and 3.5 days for OTC (SD, 3.7; minimum 2; maximum 30; median 2), was found (*P* = .05). The total number of ADD to control the outbreak was 156 in the FF group and 238 in the OTC group. Compared to metaphylaxis with a 7‐day treatment duration (ADD = 910) this represents a 64.1% and 50% reduction in antimicrobial usage for FF and OTC, respectively (*P* < .001).

### 3.4 Laboratory diagnosis

*Mycoplasma bovis*, *Pasteurella multocida*, *M. haemolytica*, *H. somni*, and PI‐3 were detected by qPCR in the nBAL samples. Both RSV and BCV were not detected. All bacterial cultures on selective indicative agar were positive for *M. bovis* and MALDI‐TOF MS identified the obtained isolate as *M. bovis*. The MIC results of the *M. bovis* strain (Table 1) indicated in vitro susceptibility of the isolate for all tested antimicrobials, including FF and OTC. Whole genome sequencing (WGS) also identified the isolate to be *M. bovis* and allowed strain classification to the Belgian genomic cluster IV.^34^

**TABLE 1:** Minimum inhibitory concentration values of the isolated *Mycoplasma bovis* from a farm facing a natural outbreak of respiratory disease, located in Flanders, Belgium

	GAM	TIL	FF	DOX	ENRO	TYL	TIA	GEN	OTC
*M. bovis*	64	>128	8	.5	.5	16	<.06	4	1
ECOFF	>64	ND	>16	>4	>2	>32	>.5	ND	>8

*Note*: Epidemiological cutoff (ECOFF) values, based on the visual estimation method as described by Bokma et al.^33^

Abbreviations: DOX, doxycycline; ENRO, enrofloxacin; FF, florfenicol; GAM, gamithromycin; GEN, gentamycin; OCT, oxytetracycline; TIA, tiamulin; TIL, tilmicosin; TYL, tylosin.

The genome of 1 of the obtained isolates was screened for previously described mutations in *M. bovis* involved in antimicrobial resistance as described elsewhere.^35^ No mutations directly associated with FF or OTC resistance were detected. However, known mutations for other antimicrobials were observed. A G748A mutation in both 23S rRNA alleles, as well as a Gln93His mutation in the *rplv* gene were found. In previous studies, G748A mutations have been observed in isolates resistant to tylosin and tilmicosin, whereas the Gln93His mutation has been linked to resistance against macrolides (gamithromycin and tylosin) when combined with a mutation in domain 5 of 1 or both 23S rRNA gene alleles on position A2058.^36^, ^37^, ^38^, ^39^ In this case, no mutations on position A2058 were found. Additionally, 1 new Lys184Asn mutation was found in the *rpId* gene. However, no links between mutations in the *rpld* gene and resistance against macrolides have been described so far. The complete *M. bovis* genome is available for download on the NCBI GenBank database under accession number CP076229.

## 4 DISCUSSION

Our main objective was to determine whether stopping antimicrobial treatment after complete reaeration of a previously consolidated lung area is an option to achieve adequate cure with less antimicrobial use. After lung reaeration, no animals relapsed in the 14‐day follow‐up period and in the months after, the farmer did not report decreased carcass weights compared to other groups of animals. However, because ultrasonographic follow‐up was not performed after day 14, a relapse with subclinical pneumonia cannot be excluded. Because of the bovine lung anatomy, with few collateral bronchi, a general assumption is that inflammation of a main bronchus more rapidly results in a consolidation of the complete lung lobe in cattle, as compared to species with many collateral bronchi, such as horses.^40^, ^41^ In this case, metaphylactic treatment was started in the early stages of the outbreak and a 0.5 cm consolidation depth was used as cutoff for treatment. In these animals, only lobular pneumonia was present, which may explain the rapid reaeration after treatment. In the 12 calves with consolidation of the complete lung lobe (≥3 cm), however, complete reaeration occurred in all but 1 calf. Hence, frequently‐encountered lung consolidations in cattle in the slaughterhouse more likely represent insufficient disease detection (including subclinical presentations) and ineffective treatment, rather than an inevitable consequence of pneumonia.^42^

Second, our study aimed to determine the difference in cure rate and healing time between 2 antimicrobials commonly used for pneumonia in calves, FF and OTC. We chose these 2 antimicrobials because of their position as first and second choice in formularies of different EU countries.^21^ In *M. bovis* from Belgian herds, acquired antimicrobial resistance against macrolides is frequent, whereas it is rare for FF and OTC, similar to most reports worldwide.^33^, ^37^ To our knowledge, this study is the first randomized clinical trial on *M. bovis* that confirmed the infection and also confirmed antimicrobial susceptibility of the strain to the antimicrobials evaluated. Treatment failure is frequent in *M. bovis* infections, and it is not clear whether this outcome is a consequence of antimicrobial resistance or the ability of *M. bovis* to escape the immune response and establish a chronic infection (eg, biofilm formation). We showed that if the *M. bovis* strain is susceptible to the antimicrobial and lung consolidations are still limited in size, a high cure rate can be achieved. For both antimicrobials, average required treatment durations were generally shorter (2.5 days for FF and 3.1 days for OTC) than the currently reported 5 to 10 days.^18^ On the other hand, 5 animals did require a much longer treatment duration before reaeration occurred. In other words, personalized treatment, in this case treatment duration, ensures cure and avoids unnecessary prolongation of antimicrobial treatment. Also, absence of reaeration may be a future indicator of treatment failure, allowing a timely switch to another antimicrobial. Although promising for application in the field, the current experimental approach of repeated ultrasonographic scanning comes with practical limitations with respect to economic feasibility. Success of ultrasonographic‐guided treatment will depend on the economic impact of having a high antimicrobial use in a given country, on the feasibility (especially speed) and reliability of the ultrasonographic method, the possibility to train people (potentially also lay staff), and on the economic impact of pneumonia that is not cured. The pressure to decrease antimicrobial use depends on local legislation, but can be a very important incentive to decrease antimicrobial use. The ultrasonographic technique used in our study requires 1 to 2 minutes per animal and is easily learned by novices.^24^ After a standard treatment of 5 to 7 days, 10.2% and 5.6% of the animals still were not cured in the FF and OTC groups, respectively. This outcome means that, when a standard 7‐day treatment is applied, these animals would still lead to economic losses as previously documented.^2^, ^43^, ^44^, ^45^

Third, in our study, FF resulted in more rapid cure than OTC, resulting in less antimicrobial use (ADD = 156). This observation may contribute substantially to decreased antimicrobial use, without hampering production or animal welfare. The MIC values from the obtained nBALF isolate suggested the present *M. bovis* strain was susceptible to both FF and OTC, which was confirmed by WGS, showing the absence of any known coding genes or point mutations associated with FF and OTC resistance. The WGS and identification of AMR genes and point mutations have proved to be valuable and quick alternatives for AMR determination. Because of their bacteriostatic effect, both FF and OTC rely on the host immune system for elimination of the infection. Under conditions where the immune system is compromised, because of stress, management factors or severe disease, bacteriostatic antibiotics are assumed to be less effective than bactericidal drugs.^46^ However, because of their clinical importance in human medicine, most bactericidal antimicrobials have been considered a third choice for respiratory disease in veterinary formularies, restricting their use in food‐producing animals in the absence of a substantiated laboratory diagnosis.^20^, ^21^, ^47^

In our study, it was surprising that there was a difference in cure rate and healing time between 2 bacteriostatic antimicrobials. Although bacteriostatic and bactericidal data may provide valuable information on the potential action of antibacterial agents in vitro, it is necessary to combine this information with pharmacokinetic and pharmacodynamic information to provide more meaningful prediction of efficacy in vivo. One reason for the observed difference in cure rate might be the lipophilic nature and wide tissue distribution of FF.^48^ Immediately after IM administration of FF, high initial blood concentrations are reached, leading to a rapid initial response.^49^ Oxytetracycline has a rather short‐term effect and lower lipid solubility (when compared to doxycycline), leading to a more restricted tissue distribution and faster elimination rate.^48^ Only 1 study using TUS previously evaluated antimicrobial treatment (tulathromycin) for BRD.^50^ Compared to that study, our cure rates using both FF and OTC were very high.^50^ Because the pathogens involved and their antimicrobial susceptibility were not determined in the previous study, it is hard to determine whether this difference in cure rate is a consequence of differences between antimicrobials, antimicrobial susceptibility of the involved pathogens or treatment initiation at a lower consolidation depth cutoff in our study.

Regardless of its feasibility under field conditions, the ultrasonographic criterion of lung reaeration can be recommended for further scientific research. Our results suggest that early initiation of antimicrobial treatment improves cure rate. All animals received antimicrobials and therefore it is difficult to make any statement about whether it would have been possible in an outbreak not to have treated the animals without lung consolidation. There is a need for this knowledge because it underlies the concept of metaphylaxis.

Being a field trial during a natural outbreak our study had some limitations. First, sample size was determined by the number of animals present at the time of the outbreak. Second, being a natural outbreak field trial, for practical reasons both ultrasound operators needed to help with injecting the animals on a limited number of time points. Despite that recognizing individual animals in the setting of the trial would have been extremely difficult because of the trial organization, we cannot consider our study completely blinded. However, we believe observer bias was limited. The use of TUS allows for a more objective diagnosis of pneumonia when compared to the use of clinical signs.^15^, ^24^, ^51^, ^52^ Third, for ethical and economic reasons we could not take the risk of not treating animals that had no lung consolidation on day 1. Despite that we are confident that today metaphylaxis is still appropriate in an *M. bovis* outbreak situation, further antimicrobial reduction may be achieved by only treating animals with consolidated lung tissue. At the time of the first visit, mean consolidation depth was still limited, suggesting the trial started at the start of the outbreak. Whether an equal decrease in treatment duration can be achieved when managing a more severe outbreak should be further addressed. Whereas our study thoroughly documented antimicrobial susceptibility in *M. bovis*, an evidenced primary pathogen, unfortunately antimicrobial susceptibility was not determined in the opportunistic *Pasteurellaceae*, such as *M. haemolytica*, *P. multocida*, and *H. somni*. These opportunists often complicate *M. bovis* infections,^9^ but it is unclear whether bacterial superinfection with these bacteria occurred in our study. The sequencing and MIC determination of a single *M. bovis* isolate also could be considered a limitation, leading to possible overinterpretation of the results. In our study, the trial was performed on a closed commercial beef farm, where no new animals had been introduced into the herd. Purchase is the most important risk factor for introducing *M. bovis* into a herd.^12^ Also, once introduced, disease by *M. bovis* is spread clonally within the herd.^53^, ^54^ Therefore, we believe chances of encountering multiple strains of *M. bovis* during this outbreak to be rather limited. Finally, dosage of the antimicrobials was based on chest circumference measured at first visit and rounded to the next 10 kg. Animals were given the same dose throughout the entire trial, not taking daily growth into account. Calculating required dosage using exact body weights may be beneficial in further decreasing required treatment duration, but is difficult to achieve in every farm.

## 5 CONCLUSIONS AND CLINICAL IMPORTANCE

Lung reaeration on ultrasonography proved to be a promising criterion to discontinue antimicrobial treatment in an outbreak of respiratory disease in beef calves. Personalizing treatment duration by using repeated TUS holds potential to both rationalize antimicrobial use and limit production losses. In our study, FF resulted in a more rapid cure rate than did OTC in the early phase of a natural outbreak of *M. bovis*, potentially complicated by *Pasteurellaceae* infection.

## CONFLICT OF INTEREST DECLARATION

Authors declare no conflict of interest.

## OFF‐LABEL ANTIMICROBIAL DECLARATION

Authors declare no off‐label use of antimicrobials.

## INSTITUTIONAL ANIMAL CARE AND USE COMMITTEE (IAUC) OR OTHER APPROVAL DECLARATION

This study was conducted in compliance with the Ghent University rules of animal experiments with the approval of the university's animal experiment ethics committee.

## HUMAN ETHICS APPROVAL DECLARATION

Authors declare human ethics approval was not needed for this study.
